# Growth Hormone-Secreting Pituitary Adenoma: Dura Mater Invasion Is Not a Predictor of Acromegaly Persistence After Trans-Sphenoidal Surgery

**DOI:** 10.3390/jcm13237312

**Published:** 2024-12-02

**Authors:** Nunzia Prencipe, Emanuele Varaldo, Giuseppe Di Perna, Luca Bertero, Alessandro Maria Berton, Bianca Maria Baldassarre, Chiara Bona, Raffaele De Marco, Fabio Bioletto, Luigi Simone Aversa, Paola Cassoni, Silvia Grottoli, Francesco Zenga

**Affiliations:** 1 Division of Endocrinology, Diabetology and Metabolism, Department of Medical Sciences, University of Turin, 10126 Turin, Italy; 2 Skull Base and Pituitary Surgery Unit, “Città della Salute e della Scienza” University Hospital, 10126 Turin, Italy; 3 Pathology Unit, Department of Medical Sciences, University of Turin, 10126 Turin, Italy; 4 Division of Endocrinology, Diabetology and Metabolism, S. Croce and Carle Cuneo Hospital District, 12100 Cuneo, Italy

**Keywords:** pituitary, acromegaly, neurosurgery, dura mater, sinus cavernous, GH, IGF-1, prognosis, remission, recurrence

## Abstract

**Objectives**: In pituitary adenomas, examinations of surgical specimens have shown that dural invasion occurs in 42–85% of cases. No studies about dura mater invasion have been conducted specifically in acromegaly patients. The aim of the present study was to evaluate the relationship between histologically dural invasion and the main features of GH-secreting adenomas. **Methods**: This retrospective study included all consecutive acromegaly patients who underwent neurosurgery at our university hospital between 2017 and 2020. The following data were collected: (1) clinical, biochemical and morphological data at diagnosis, at three months, one year after neurosurgery, and at last follow-up; (2) pathological features (dura mater invasion, immunohistochemical analyses, proliferation index Ki-67, p53, and granulation pattern); and (3) radiological features on magnetic resonance images. **Results**: Of 35 acromegaly patients, 11 had dural invasion (INV+ 31%) and 24 did not (INV− 69%). GH levels at diagnosis were greater in INV+ patients (*p* = 0.02), and a GH value > 27 ng/mL was able to distinguish INV+ patients (Sensitivity 80%, Specificity 73%, AUC 0.760, *p* = 0.006). Indeed, patients with GH > 27 ng/mL at diagnosis had a tenfold greater risk of dura mater invasion (OR 10.7; 95% CI 1.74–65.27, *p* = 0.005). No differences were found in the other clinical, biochemical, morphological, radiological and pathological features. Regarding remission likelihood, IGF-1 levels at diagnosis were lower in cured patients (*p* = 0.03). **Conclusions**: The GH level at diagnosis is the only parameter significantly associated with dura mater invasion. Lower IGF-1 levels at diagnosis are significantly associated with remission one year after surgery.

## 1. Introduction

Acromegaly is a rare disease with an estimated incidence of 3–4 new cases per million people per year, and its prevalence has been increasing in recent decades [1,2]. It is caused by a chronic excess of growth hormone (GH) and insulin-like growth factor 1 (IGF-1), in most cases as a result of a sporadic GH-secreting pituitary tumor. Current treatment modalities include surgery, medical therapy and radiotherapy, and according to the most recent guidelines, the surgical removal of pituitary tumors is considered the first-line approach [3,4]. Despite the benign nature of pituitary adenomas, regardless of their secretion type, the microscopic examination of surgical specimens has shown that the dural invasion of pituitary adenomas occurs in approximately 42–85% of cases [5,6]. Several authors have reported that the rate of dural invasion is higher in large adenomas compared to smaller ones [6]. In contrast, Knosp et al. concluded that invasion is not related to adenoma size but is instead associated with disease recurrence and patient age [7].

In general, poorly differentiated (e.g., plurihormonal Pit-1-positive), sparsely granulated (SG), and hormonally inactive pituitary adenomas tend to be more invasive than well-differentiated, densely granulated (DG), and hormonally active adenomas [8,9]. However, Selman et al. reported no evidence of a correlation between invasiveness and the immunohistochemical classification of tumor type [6]. The ability of pituitary adenomas to infiltrate the dura may contribute to their persistence or recurrence after neurosurgery, potentially affecting the cure rate for hormonally active tumors and the recurrence rate for clinically nonfunctioning adenomas [6,8].

In the early 1940s, invasive pituitary adenomas were defined as those extending beyond their capsules and invading adjacent structures [10]. Over time, the definition of aggressive pituitary tumors has evolved considerably. The 2014 WHO classification defined adenomas exhibiting histological features suggestive of aggressive clinical behavior (including an elevated mitotic index, a Ki-67-labelling index greater than 3% and overexpression of the p53 protein according to immunohistochemistry) as “atypical”. However, in the current WHO classification [11], the term “atypical adenoma” is no longer recommended. Instead, a thorough clinical evaluation of aggressiveness is strongly recommended on a case-by-case basis, although accurate tumor subtyping (including an assessment of the tumor proliferative potential by mitotic count and Ki-67 index, as well as MRI findings and/or intraoperative impression) remains essential [11]. In addition, a new five-tiered classification has been proposed, taking into account invasion, immunohistochemical (IHC) type, and proliferative markers (Ki-67 index, mitotic count, p53 positivity) for prognostic and clinical scoring [12].

To our knowledge, no studies specifically addressing dura mater invasion in the acromegaly population have been conducted. This considered, the aim of the present study was to evaluate the relationship between histologically verified dural invasion and the proliferative potential of GH-secreting pituitary adenomas. In particular, the primary outcome was to correlate dura mater invasion with biochemical and morphological remission, persistence, or recurrence of acromegaly disease. The secondary outcomes were to correlate dural invasion with proposed predictors of more aggressive disease and first-generation somatostatin receptor ligand (SRL) responsiveness/resistance.

## 2. Materials and Methods

The present retrospective study included all consecutive acromegaly patients who underwent pituitary neurosurgery at the university hospital “Città della Salute e della Scienza di Torino” (Turin, Italy) between December 2017 and December 2020. A “two nostrils-four hands” endoscopic endonasal approach using a 3D-HD rigid endoscope (Visionsense Ltd., Petah Tikva, Israel) was performed in all cases by the same experienced neurosurgeon (FZ), with an annual caseload of more than 50 pituitary surgeries [13,14]. No exclusion criteria were applied, except for patients in whom dura mater samples could not be obtained during surgery.

For each patient, the following data were collected: (1) clinical, biochemical and morphological data at diagnosis, three months, one year after neurosurgery and at last follow-up; (2) GH levels on the first (GH1) and second postoperative day (GH2); (3) pathological features including dura mater invasion, IHC analysis (Ki-67 proliferation index, p53 positivity and granulation pattern according to the CAM5.2 staining pattern); and (4) radiological findings from MRI scans, focusing on T2-weighted image intensity and Knosp grade. Additionally, where possible, the Trouillas’ grading system score was calculated, based on both local cavernous sinus invasiveness and proliferation criteria [12].

Acromegaly was biochemically diagnosed by fasting plasma IGF-1 concentrations above the normal ranges for age, together with failure to suppress serum GH concentrations below 1 ng/mL after a 75 g oral glucose tolerance test (OGTT), following international guidelines [3]. After surgery, all patients were re-evaluated, both through IGF-1 levels and OGTT at three months and through IGF-1 levels after one year—this was our clinical practice. Remission was defined by a GH level during the OGTT of less than 1 ng/mL at three months, along with an age-adjusted normal IGF-1 level at both three months and one year post-surgery, without any medical treatment.

All the data were extracted either from a recorded online and shared database or collected during patient hospitalization and follow-up. The data included demographic characteristics, pre- and postoperative hormonal data, radiological findings from pre- and postoperative MRI, tumor histology and specific details of the surgical approach performed.

### 2.1. Determination Methods

Serum GH levels (ng/mL) were measured in duplicate by the immunoradiometric assay (IRMA) method (IRMA GH, Beckman Coulter, Chodov, Czech Republic). The sensitivity of the assay was 0.033 ng/mL, and the inter- and intra-assay coefficients of variation (CVs) were 9.0–14.0% and 2.4–6.5%, respectively. Serum IGF-I levels (ng/mL) were measured in duplicate by the radioimmunoassay (RIA) method (SM-C RIA-CT, DIA source ImmunoAssays, Ottignies-Louvain-la-Neuve, Belgium) after acid-ethanol extraction to avoid interference from binding proteins. The sensitivity of the method was 0.25 ng/mL, and the inter- and intra-assay CVs were 6.8–14.9% and 4.5–7.0%, respectively. Considering the variability in normal values based on patients’ age, IGF-I levels were additionally standardized according to the upper limit of normality (ULN).

### 2.2. Collection and Analysis of the Dural Specimen

A classical endoscopic endonasal approach was used [13,14]. Once the sphenoid sinus was entered, the posterior wall was exposed by removing the mucosa, and the anatomical landmarks were identified. The sellar floor was opened “from blue to blue”, exposing the medial wall of the cavernous sinus bilaterally. Then, the dura mater was cross-cut, and it was carefully dissected from the underlying tumor pseudo-capsule that was preserved in order to reduce tumoral cells spreading. After dissection, a triangular-shaped piece of dura mater was cut and sent to a pathologist to analyze tumoral invasion. Particular attention was paid when the incision was made toward the medial wall of the cavernous sinus due to the increased risk of venous bleeding. However, when bleeding occurred, hemostasis was adequately achieved with a thrombin matrix, gelfoam and compression.

**Figure 1:**
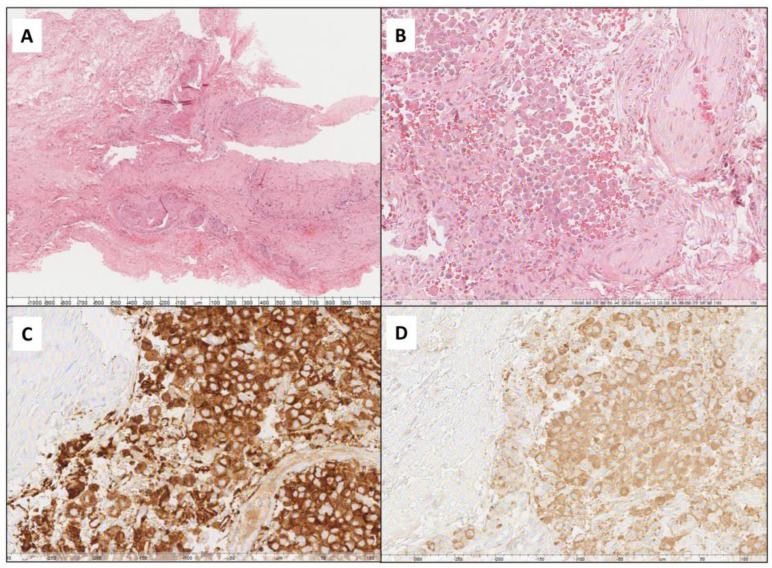
Histological images showing a meningeal sample without infiltration ((**A**) hematoxylin/eosin, original magnification: 40×) and one infiltrated by somatotroph pituitary adenoma ((**B**) hematoxylin/eosin, original magnification: 200×; (**C**) synaptophysin immunohistochemistry, original magnification: 200×; (**D**) GH immunohistochemistry, original magnification: 200×).

Dura mater invasion was assessed by the histopathological examination of conventional hematoxylin-eosin (H&E)-stained sections. If the morphological assessment was uncertain, synaptophysin IHC was performed (Figure 1).

All the subjects provided informed consent for the processing of their data. The study was approved by the local Ethics Committee (cod. 0040828) and was in accordance with the principles of the Declaration of Helsinki.

### 2.3. Statistical Analysis

Normally and non-normally distributed variables were expressed as mean and standard deviation (SD) or median and interquartile range (IQR), respectively, while categorical data were expressed as counts and percentages. Normality was assessed using the Shapiro–Wilk test.

Between-group differences were evaluated by the Student’s t-test or the Mann-Whitney U test for continuous variables and the chi-square test or Fisher’s exact test for categorical variables. A multivariate logistic regression model was constructed to predict dural invasion. Receiver operating characteristics (ROC) analysis was used to assess the cut-offs for GH, with maximum sensitivity and specificity, in predicting dural infiltration (Youden index). Univariate logistic regression analysis was performed to determine any predictive factors of acromegaly remission. A cut-off of *p*-value < 0.05 was considered statistically significant. Statistical analysis was performed using the computing environment MedCalc^®^ (Statistical Software version 18.11.3, MedCalc Software Ltd., Ostend, Belgium).

## 3. Results

Thirty-five acromegaly treatment-naïve patients (46% male and 54% female; mean age at diagnosis 53.4 ± 13.9 years and mean age at surgery 55.1 ± 13.7 years) were enrolled in the study. At diagnosis, 77% of patients had a macroadenoma, and 20% had an impaired visual field.

We divided patients into two groups based on whether they had (INV+) or did not have (INV−) dural invasion, according to the pathological analysis. Eleven patients had dural invasion (31%), while twenty-four did not (69%). No significant differences were found in sex (*p* = 1.00) or age at diagnosis (*p* = 0.15) between the two groups. The main characteristics of the two groups are summarized in Table 1.

No significant difference was found between INV+ and INV− groups in IGF-1 levels (752 [548–987] ng/mL vs. 664 [394–894] ng/mL, *p*= 0.44) or IGF-1/ULN (2.4 [1.68–3.1] vs. 2.5 [2.3–3.3], *p* = 0.57). In contrast, random GH levels at diagnosis were higher in INV+ subjects (84.5 [29.0–153.0] ng/mL vs. 17.2 [4.4–36.0] ng/mL, *p* = 0.02). However, GH nadir levels after OGTT at diagnosis, available for 22 patients, showed no significant differences between the INV+ and INV− groups (3.7 [1.8–3.8] ng/mL vs. 2.5 [1.4–8.0] ng/mL, *p* = 0.91).

ROC analysis for GH levels at diagnosis showed that GH > 27 ng/mL distinguished patients with dura mater invasion (Sensitivity 80%*,* Specificity 73%*,* AUC 0.760, *p* = 0.006). Indeed, logistic regression analysis revealed that patients with GH levels > 27 ng/mL at diagnosis had a tenfold greater risk of dural invasion than the other patients (OR 10.7; 95% CI 1.74–65.27, *p* = 0.005). These results also remained significant in the multivariate analysis, stratifying for tumor diameter, Ki-67 expression, age at diagnosis and surgery, cavernous sinus invasion and granulation pattern (Table 2).

The adenoma dimensions did not differ significantly between the two groups, considering the larger (*p* = 0.11) and the smaller (*p* = 0.55) diameter. The rate of dural invasion was evaluated according to tumor diameter, categorized as D1 (≤10 mm), D2 (11–20 mm) and D3 (≥21 mm). At D1, the invasion rate was 22%; at D2, it was 25%; and at D3, it reached 67%. Although there was a greater invasion rate in larger tumors, this difference was not statistically significant (χ^2^ test, *p* = 0.12). In 30 patients, it was possible to obtain the Trouillas score: 40% were classified in group 1a, 20% were classified in group 1b, 33% were classified in group 2a and 17% were classified in group 2b, with no significant difference between INV+ and INV− patients (*p* = 0.196).

No difference was found in the intensity of the T2-weighted MRI images (INV+ vs. INV−: hypointensity: 50% vs. 35%; isointensity: 40% vs. 25%; hyperintensity: 10% vs. 40%); or in the following invasion parameters: visual field impairment (19% INV+ vs. 21% INV−), suprasellar extension (55% vs. 42%), cavernous sinus invasion (33% vs. 58%) and Knosp grading (grade 0: 40% vs. 55%, grade 1: 20% vs. 28%, grade 2: 20% vs. 11%, grade 3: 20% vs. 6%).

### 3.1. Perioperative Results

The mean surgery time was 94 ± 24 min, with no significant difference between the INV+ and INV− patients (99 ± 25 min vs. 92 ± 23 min). Both GH1 levels (1.6 [1.2–3] ng/mL vs. 1.2 [0.7–2.4] ng/mL, *p* = 0.24) and GH2 levels (1.7 [0.8–4.1] ng/mL vs. 0.9 [0.6–1.3] ng/mL, *p* = 0.17) were comparable between the two groups.

### 3.2. Pathological Features

Considering the entire study population, eight patients exhibited immunohistochemistry positivity only for GH, while twenty-four patients showed positivity for multiple hormones (20/24 for prolactin [PRL]). Additionally, three patients had a negative IHC analysis for GH despite a biochemical diagnosis of the disease (however, in these patients, PIT-1 staining was positive). In the INV+ group, only three patients were positive solely for GH, compared to five patients in the INV− group (*p* = 0.820). No significant differences were found in the granulation pattern between the two groups (INV+ vs. INV−; SG/DG 44% vs. 50%), the Ki-67 index (Ki-67 ≥ 3%: 27% vs. 37%), or p53 positivity.

### 3.3. Analysis at Follow-Up (3 and 12 Months After Surgery and at the Last Visit)

Three months post-surgery, IGF-1 levels (269 [136–458] ng/mL vs. 213 [157–275] ng/mL), the GH nadir during OGTT (0.4 [0.1–0.63] ng/mL vs. 0.2 [0.1–1.37] ng/mL), and the remission rate were similar between the two groups (INV+ vs. INV−: 73% vs. 88%). A tumor remnant was observed in 46% of patients with dural invasion and in 38% of patients without invasion. At the one-year follow-up, data were available for 20 patients. IGF-1 levels (191.9 ± 66.4 ng/mL vs. 233.6 ± 89.0 ng/mL, *p* = 0.29) and the remission rate (36% vs. 53%, *p* = 0.74) were also similar between the two groups (Table 3).

At the last follow-up (50.4 ± 10.9 months after surgery), data were available for 18 patients (51.4% of the starting sample). Of these, five patients (28%) had active disease, while thirteen (72%) were in persistent remission, and no significant difference in dural invasion was found (*p* = 0.63). A tumor remnant was present in seven patients (two in the active group and five in the remission group). Only one patient who showed biochemical remission at the 12-month follow-up presented disease recurrence afterward.

### 3.4. Predictive Parameters of Remission

**Figure 2:**
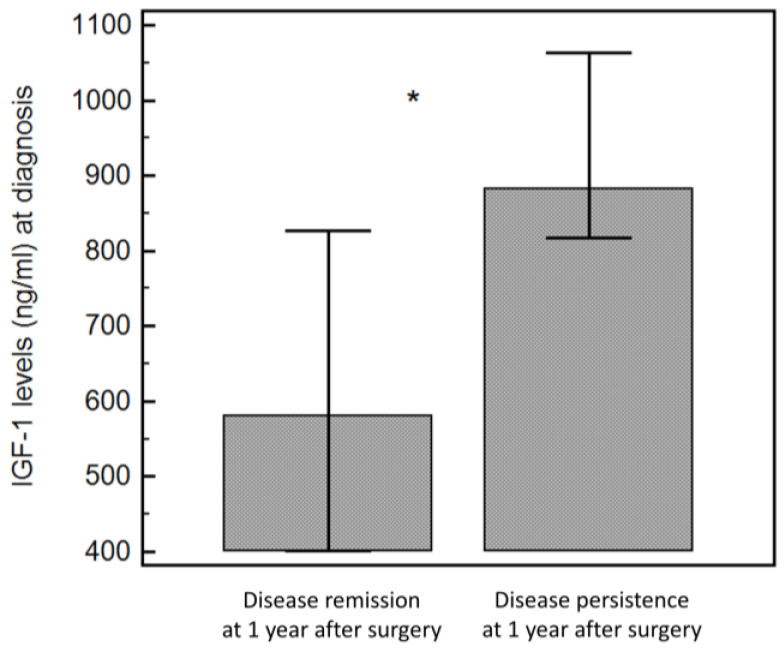
Difference in insulin-like growth factor 1 (IGF-1) levels between patients with disease remission and those with persistent disease at 1 year after surgery. * = *p* < 0.05.

IGF-1 levels at diagnosis (581.0 [422.5–787.2] ng/mL vs. 883.0 [832.0–1028.0] ng/mL, *p* = 0.006) were the only parameter significantly lower in patients with disease remission at one year, although the significance was borderline when adjusted for ULN (2.3 [1.8–2.6] vs. 2.7 [2.5–3.2], *p* = 0.07) (Figure 2).

In our population, dural invasion was not found to be a predictor of remission at the last follow-up, nor were age at diagnosis, age at surgery, adenoma diameter, IGF-1 levels and IGF-I/ULN ratio at diagnosis, cavernous sinus invasion, Ki-67 index and signal intensity on T2-weighted MRI sequences.

## 4. Discussion

For the first time, our study evaluated the role of dura mater invasion as a predictor of the persistence of acromegaly in a homogeneous series of patients with GH-secreting pituitary adenomas. While some previous studies [6,15,16,17] have investigated the incidence of dura mater invasion in pituitary adenomas, they did not focus on a homogenous series of GH-secreting adenomas. Furthermore, while these studies have provided valuable insights into the role of dura mater invasion, their findings were limited by methodological constraints, including non-consecutive sampling and a predominance of macroadenomas. Notably, no study to date has investigated the prognostic significance of dura mater invasion specifically in patients with acromegaly.

In our study, the histological invasion of the dura mater was demonstrated in 31% of acromegalic patients, which is in line with the findings of Meij et al. [17], who documented the invasion of the dura mater in 33.8% of pituitary adenomas with GH and/or GH/PRL positivity.

A particularly interesting result concerns the GH levels at diagnosis, which were significantly higher in patients with dura mater invasion. Additionally, a cut-off of 27 ng/mL was found as the best parameter for differentiating the two populations. In fact, patients with GH concentrations > 27 ng/mL at diagnosis exhibited over a tenfold increased risk of dura mater invasion, suggesting that lesions with higher GH levels could be more aggressive. It is known that GH levels are positively correlated with disease severity, which tends to be more difficult to manage. Kim H.J. et al. reported significantly higher random GH and post-OGTT GH levels in patients with persistent acromegaly [18]. The significant difference in GH levels at diagnosis may be attributable to the larger size of adenomas invading the dura mater. Meij et al. [17] highlighted a direct correlation between tumor size and dura mater invasion, noting that 24% of patients with microadenomas exhibited dura invasion, while up to 70% of patients with macroadenomas did. In our study, however, we demonstrated that tumor size, cavernous sinus invasion, age at diagnosis, Ki-67 index, and granulation pattern did not significantly influence the association between dura mater invasion and elevated GH levels at diagnosis.

In addition, considering that GH levels on the first postoperative day are a highly specific predictor for the early diagnosis of long-term acromegaly persistence [19,20], we analyzed the role of GH levels on the first and second postoperative day without detecting any difference in subjects with or without dural invasion, in contrast to the findings at diagnosis.

Although the role of GH (at diagnosis) as a predictor of dura mater invasion is evident in our study, the same cannot be said for IGF-1 levels at diagnosis or the IGF-1/ULN ratio. The discrepancy between GH and IGF-1 in predicting dural invasion allows us to hypothesize that GH may exert autocrine- or paracrine-stimulating effects on the pituitary tumor itself. In fact, an autocrine action of GH has been demonstrated in endometrial carcinoma [21] and in breast cancer cells [22]; moreover, GH promotes invasion and the migration of tumor cells [22]. In the literature, however, there is no clear evidence of the direct GH stimulation of pituitary tumors. In an autopsy series, Mertani et al. [23] demonstrated the expression of both the protein and mRNA of the GH receptor in healthy pituitary glands, suggesting direct paracrine, autocrine, or intracrine effects of GH on pituitary cells. Similarly, it is possible to hypothesize that paracrine stimulation by GH-secreting pituitary tumors leads to proliferation and the invasiveness of the tumor itself.

We also evaluated whether other predictors of dura mater invasion were already present at diagnosis. In this regard, an MRI of the sellar region is not able to accurately highlight the invasion of the dura mater by the tumor, as it cannot effectively identify the medial wall of the dura mater of the cavernous sinus [24]. Among the morphological parameters that can be analyzed by MRI, neither the suprasellar nor parasellar extension of the tumor, nor the Knosp grade or intensity of the adenoma on MRI (particularly hyperintensity on T2-weighted images), were good predictors of dura mater invasion in our series.

Finally, focusing on the histological granular pattern, no differences in dura mater invasion between sparsely and densely granulated adenomas were demonstrated, as previously described [17]. In contrast to other published data [17], we did not find a significant difference in dura mater invasion concerning tumor size. However, it should be noted that the GH-secreting adenomas invading the dura mater were larger in our study, although we could not establish a statistically significant difference. The same previous work revealed a significant correlation between dura mater invasion and adenoma size when tumors were classified into arbitrary categories (less than 10 mm, between 10 mm and 20 mm, between 20 mm and 40 mm, and greater than 40 mm) [17]. This difference was also present in our study, although not statistically significant, showing a clear positive trend. For adenomas smaller than 10 mm, the invasion rate was 22%; for those between 10 and 20 mm, it was 25%; and for those larger than 20 mm, it was 67%.

Unlike previous studies, neither the age at diagnosis nor the age at the time of surgery showed a significant difference in our cohort. Meij et al. [17] reported that the incidence of dura mater invasion increases with age, attributing this difference to the fact that older patients have larger lesions due to delayed diagnosis. While this may hold true for non-secreting adenomas, it appears that GH-secreting adenomas tend to be more aggressive and larger in younger patients. Taghvaei et al. [25] demonstrated that young acromegalic patients have more aggressive pituitary lesions, characterized by a higher proliferation index and increased p53 expression, as well as a lower probability of remission following transsphenoidal adenomectomy.

These data were also supported by our study, although we could not demonstrate a statistically significant difference. The age at diagnosis was lower in patients with dura mater invasion, leading us to hypothesize that younger patients may have more invasive disease. Additionally, regarding peri- and postoperative complications, our study found no differences between the two groups, which aligns with findings previously reported in the literature.

This is the first study to examine the predictive role of dura mater invasion specifically in patients with GH-secreting adenomas with respect to disease persistence or remission after surgery. The currently available data do not reveal clear differences between patients with and without dura mater invasion. In particular, the rate of disease remission three months after neurosurgery was comparable between patients with or without dura invasion, as was the percentage of patients in remission one year after surgery. Regardless, these findings require some reflection.

First, the sample size was likely not sufficient to identify significant differences, although this was expected considering the low incidence of acromegaly (0.2–1.1 cases per 100,000 individuals per year) [3]. Moreover, the follow-up period was rather short, and some patients were lost to follow-up. In this context, Meij et al. [17] reported a slight but significant difference in the survival rate between invasive (91%) and noninvasive (100%) adenomas over a mean observation period of six years. Therefore, a longer follow-up is certainly necessary to evaluate long-term disease remission and, more importantly, survival rates.

A secondary focus of our study was to evaluate the presence of predictors of recovery from acromegalic disease one year after surgery. In our series, remission at one year was achieved by 11 out of 20 patients, representing 55% of the study population—a percentage fully comparable to that reported in the literature. Starnoni et al. [26], in fact, in their meta-analysis published in 2016 (evaluating 1,105 acromegalic patients), reported a cure rate of approximately 55% (95% CI 44.4–65.2%). Although invasion of the dura mater did not prove to be a significant predictor of recovery during the postoperative period, we highlighted interesting differences between patients who achieved remission and those with persistent disease. First, IGF-1 levels at diagnosis were lower in the former group, although this difference did not reach significance when evaluating the IGF-1/ULN ratio, even if the result was borderline significant. The other hormonal parameters (random GH evaluation and nadir after OGTT) were not different, contrary to the findings of other studies [18]. Similarly, for all the pathological parameters, we found no significant difference between the two groups, unlike the study by Katja Kiseljak-Vassiliades et al. [27], which revealed a significant difference in remission between sparsely granulated and densely granulated adenomas. Finally, no significant differences were found in neuroimaging parameters.

Despite some of the limitations mentioned (e.g., small sample size, limited follow-up), our work has several strengths. Unlike other retrospective and multicenter studies, it benefits from the reproducibility of dura mater specimens (all collected by the same neurosurgeon, an expert in pituitary surgery) and the use of standardized laboratory methods and kits for biochemical and immunohistochemical analyses.

In conclusion, this single-center study investigated the role of dura mater invasion as a predictor of disease remission or persistence in patients with GH-secreting adenomas who underwent neurosurgery. It highlighted that dura mater invasion does not affect the likelihood of recovery from acromegaly at 12 months. The incidence of dura mater invasion in patients with GH-secreting adenomas was 31%, which is in line with the incidence reported in other studies in the literature. The only parameter significantly associated with dura mater invasion by the pituitary lesion was GH levels at diagnosis. Additionally, we evaluated predictors of remission from acromegalic disease 12 months after neurosurgery. The data confirmed that lower plasma IGF-1 levels at diagnosis are significantly associated with remission after resection surgery.

## Figures and Tables

**Table 1:** Clinical features at diagnosis in patients with (INV+) and without (INV−) dura mater invasion.

Variable	INV+ (*N* = 11)	INV− (*N* = 24)	*p*-Value
Sex, Female; n (%)	6 (55)	13 (54)	1.00
Age at diagnosis, years; mean ± SD	48.5 ± 17.6	55.7 ± 11.5	0.15
Age at neurosurgery, years; mean ± SD	50.6 ± 18.3	57.1 ± 10.9	0.20
Macroadenoma; n (%)	9 (82)	18 (75)	1.00
Greater diameter, mm; mean ± SD	15.9 ± 7.1	12 ± 5.4	0.11
Smaller diameter, mm; mean ± SD	8.2 ± 3.4	8.6 ± 5.0	0.55
Visual field impairment; n (%)	2 (18)	5 (21)	1.00
GH levels, ng/mL; median [IQR]	84.5 [29.0–153.0]	17.2 [4.4–36.0]	**0.02**
GH nadir post-OGTT, ng/mL; median [IQR] *	3.7 [1.8–3.8]	2.5 [1.4–8.0]	0.91
IGF-1 levels, ng/mL; median [IQR]	752 [548–987]	664 [394–894]	0.44
IGF-1/ULN ratio; median [IQR]	2.4 [2.3–2.9]	2.5 [1.8–2.9]	0.57

The numbers in bold indicate significant values (*p* < 0.05). * Available in 22 subjects. Abbreviations: SD: standard deviation; IQR: interquartile range; GH: growth hormone; IGF-1: insulin-like growth factor 1; ULN: upper limit of normal.

**Table 2:** Logistic regression models evaluating the association between growth hormone (GH) levels at diagnosis > 27 ng/mL and other variables indicative of tumor aggressiveness in predicting dura mater invasion.

Outcome	Predictor	Model Adjustment	Odds Ratio	95% CI	*p*-Value
Dura mater invasion	GH levels at diagnosis > 27 ng/ml	Adenoma max diameter	9.9	1.5–65.4	**0.02**
		Ki-67 ≥ 3%	10.8	1.7–67.0	**0.01**
		Sinus cavernous invasion	10.73	1.53–75	**0.02**
		Sparsely granulated pattern	7.4	1.1–49.1	**0.04**
		Age at diagnosis	17.39	2.1–145.5	**0.008**
		Age at surgery	10.03	1.3–77.7	**0.02**

The numbers in bold indicate significant values (*p* < 0.05). Abbreviations: GH: growth hormone; CI: confidence interval.

**Table 3:** Clinical and biochemical features three months and one year after neurosurgery in patients with (INV+) and without (INV−) dura mater invasion.

Variable Evaluated at 3 Months	INV+ (*N* = 11)	INV− (*N* = 24)	*p*-Value
Residual tumor; n (%)	5 (46)	9 (38)	0.70
IGF-1 levels, ng/mL; median [IQR]	269.0 [136.0–458.0]	213.0 [157.5–275.5]	0.27
IGF-1/ULN ratio; median [IQR]	0.8 [0.6–1.2]	0.8 [0.7–1.0]	0.90
GH nadir post-OGTT, ng/mL; median [IQR]	0.4 [0.1–0.6]	0.2 [0.1–1.4]	0.80
Remission rate; n (%)	8 (73)	21 (88)	0.35
**Variable evaluated at 1 year**	**INV+ (*N* = 7)**	**INV− (*N* = 13)**	***p*-Value**
IGF-1 levels, ng/mL; median [IQR]	191.9 ± 66.4	233.6 ± 89.0	0.29
IGF-1/ULN ratio; median [IQR]	0.7 [0.4–0.8]	0.8 [0.6–0.9]	0.30
Remission rate; n (%)	4 (36)	7 (53)	0.74
**Variable evaluated at last follow-up**	**INV+ (*N* = 7)**	**INV− (*N* = 11)**	***p*-Value**
IGF-1 levels, ng/mL; median [IQR]	159.0 [143.7–225.7]	187.0 [152.2–255.2]	0.38
IGF-1/ULN ratio; median [IQR]	0.8 [0.7–0.9]	0.8 [0.7–1.0]	0.87
Remission rate; n (%)	6 (86)	7 (74)	0.63

Abbreviations: IGF-1: insulin-like growth factor 1; GH: growth hormone; OGTT: oral glucose tolerance test; IQR: interquartile range.

## Data Availability

The datasets generated during and/or analyzed during the current study are not publicly available but are available from the corresponding author upon reasonable request.

## References

[1] Cannavò S., Ferraù F., Ragonese M., Curtò L., Torre M.L., Magistri M., Marchese A., Alibrandi A., Trimarchi F. (2010). Increased Prevalence of Acromegaly in a Highly Polluted Area. Eur. J. Endocrinol..

[2] Caputo M., Ucciero A., Mele C., De Marchi L., Magnani C., Cena T., Marzullo P., Barone-Adesi F., Aimaretti G. (2019). Use of Administrative Health Databases to Estimate Incidence and Prevalence of Acromegaly in Piedmont Region, Italy. J. Endocrinol. Investig..

[3] Katznelson L., Laws E.R., Melmed S., Molitch M.E., Murad M.H., Utz A., Wass J.A.H. (2014). Acromegaly: An Endocrine Society Clinical Practice Guideline. J. Clin. Endocrinol. Metab..

[4] Giustina A., Biermasz N., Casanueva F.F., Fleseriu M., Mortini P., Strasburger C., van der Lely A.J., Wass J., Melmed S. (2024). Acromegaly Consensus Group Consensus on Criteria for Acromegaly Diagnosis and Remission. Pituitary.

[5] Sautner D., Saeger W. (1991). Invasiveness of Pituitary Adenomas. Pathol. Res. Pract..

[6] Selman W.R., Laws E.R., Scheithauer B.W., Carpenter S.M. (1986). The Occurrence of Dural Invasion in Pituitary Adenomas. J. Neurosurg..

[7] Knosp E., Kitz K., Steiner E., Matula C. (1991). Pituitary Adenomas with Parasellar Invasion. Acta Neurochir. Suppl..

[8] Scheithauer B.W., Kovacs K.T., Laws E.R., Randall R.V. (1986). Pathology of Invasive Pituitary Tumors with Special Reference to Functional Classification. J. Neurosurg..

[9] Bioletto F., Berton A.M., Prencipe N., Varaldo E., Bona C., Grottoli S. (2022). Markers of Aggressiveness in Pituitary Tumors: Update and Perspectives. J. Clin. Med..

[10] Jefferson G. (1940). Extrasellar Extensions of Pituitary Adenomas: (Section of Neurology). Proc. R Soc. Med..

[11] Mete O., Lopes M.B. (2017). Overview of the 2017 WHO Classification of Pituitary Tumors. Endocr. Pathol..

[12] Trouillas J., Jaffrain-Rea M.-L., Vasiljevic A., Raverot G., Roncaroli F., Villa C. (2020). How to Classify the Pituitary Neuroendocrine Tumors (PitNET)s in 2020. Cancers.

[13] Di Perna G., Penner F., Cofano F., De Marco R., Baldassarre B.M., Portonero I., Garbossa D., Ceroni L., Pecorari G., Zenga F. (2021). Skull Base Reconstruction: A Question of Flow? A Critical Analysis of 521 Endoscopic Endonasal Surgeries. PLoS ONE.

[14] Pennacchietti V., Garzaro M., Grottoli S., Pacca P., Garbossa D., Ducati A., Zenga F. (2016). Three-Dimensional Endoscopic Endonasal Approach and Outcomes in Sellar Lesions: A Single-Center Experience of 104 Cases. World Neurosurg..

[15] Daita G., Yonemasu Y. (1996). Dural Invasion and Proliferative Potential of Pituitary Adenomas. Neurol Med. Chir..

[16] Lonser R.R., Ksendzovsky A., Wind J.J., Vortmeyer A.O., Oldfield E.H. (2012). Prospective Evaluation of the Characteristics and Incidence of Adenoma-Associated Dural Invasion in Cushing Disease. J. Neurosurg..

[17] Meij B.P., Lopes M.-B.S., Ellegala D.B., Alden T.D., Laws E.R. (2002). The Long-Term Significance of Microscopic Dural Invasion in 354 Patients with Pituitary Adenomas Treated with Transsphenoidal Surgery. J. Neurosurg..

[18] Kim J.H., Hur K.Y., Lee J.H., Lee J.H., Se Y.-B., Kim H.I., Lee S.H., Nam D.-H., Kim S.Y., Kim K.-W. (2017). Outcome of Endoscopic Transsphenoidal Surgery for Acromegaly. World Neurosurg..

[19] Cambria V., Beccuti G., Prencipe N., Penner F., Gasco V., Gatti F., Romanisio M., Caputo M., Ghigo E., Zenga F. (2021). First but Not Second Postoperative Day Growth Hormone Assessments as Early Predictive Tests for Long-Term Acromegaly Persistence. J. Endocrinol. Investig..

[20] Varaldo E., Prencipe N., Berton A.M., Aversa L.S., Bioletto F., De Marco R., Gasco V., Zenga F., Grottoli S. (2024). Utility of Copeptin in Predicting Non-Pathological Postoperative Polyuria in Patients Affected by Acromegaly Undergoing Pituitary Neurosurgery. Pituitary.

[21] Pandey V., Perry J.K., Mohankumar K.M., Kong X.-J., Liu S.-M., Wu Z.-S., Mitchell M.D., Zhu T., Lobie P.E. (2008). Autocrine Human Growth Hormone Stimulates Oncogenicity of Endometrial Carcinoma Cells. Endocrinology.

[22] Chen Y.-J., Zhang X., Wu Z.-S., Wang J.-J., Lau A.Y.-C., Zhu T., Lobie P.E. (2015). Autocrine Human Growth Hormone Stimulates the Tumor Initiating Capacity and Metastasis of Estrogen Receptor-Negative Mammary Carcinoma Cells. Cancer Lett..

[23] Mertani H.C., Pechoux C., Garcia-Caballero T., Waters M.J., Morel G. (1995). Cellular Localization of the Growth Hormone Receptor/Binding Protein in the Human Anterior Pituitary Gland. J. Clin. Endocrinol. Metab..

[24] Scotti G., Yu C.Y., Dillon W.P., Norman D., Colombo N., Newton T.H., De Groot J., Wilson C.B. (1988). MR Imaging of Cavernous Sinus Involvement by Pituitary Adenomas. AJR Am. J. Roentgenol..

[25] Taghvaei M., Sadrehosseini S.M., Ardakani J.B., Nakhjavani M., Zeinalizadeh M. (2018). Endoscopic Endonasal Approach to the Growth Hormone-Secreting Pituitary Adenomas: Endocrinologic Outcome in 68 Patients. World Neurosurg..

[26] Starnoni D., Daniel R.T., Marino L., Pitteloud N., Levivier M., Messerer M. (2016). Surgical Treatment of Acromegaly According to the 2010 Remission Criteria: Systematic Review and Meta-Analysis. Acta Neurochir..

[27] Kiseljak-Vassiliades K., Carlson N.E., Borges M.T., Kleinschmidt-DeMasters B.K., Lillehei K.O., Kerr J.M., Wierman M.E. (2015). Growth Hormone Tumor Histological Subtypes Predict Response to Surgical and Medical Therapy. Endocrine.

